# Lignicolous freshwater ascomycota from Thailand: Phylogenetic and morphological characterisation of two new freshwater fungi: *Tingoldiago hydei* sp. nov. and *T. clavata* sp. nov. from Eastern Thailand

**DOI:** 10.3897/mycokeys.65.49769

**Published:** 2020-03-26

**Authors:** Li Xu, Dan-Feng Bao, Zong-Long Luo, Xi-Jun Su, Hong-Wei Shen, Hong-Yan Su

**Affiliations:** 1 College of Basic Medicine, Dali University, Dali 671003, Yunnan, China Dali University Dali China; 2 College of Agriculture & Biological Sciences, Dali University, Dali 671003, Yunnan, China Mae Fah Luang University Chiang Rai Thailand; 3 Center of Excellence in Fungal Research, Mae Fah Luang University, Chiang Rai 57100, Thailand Chiang Mai University Chiang Mai Thailand; 4 Department of Entomology & Plant Pathology, Faculty of Agriculture, Chiang Mai University, Chiang Mai 50200, Thailand Mae Fah Luang University Chiang Mai Thailand

**Keywords:** 2 new species, Lentitheciaceae, Freshwater fungi, phylogeny, taxonomy

## Abstract

Lignicolous freshwater fungi represent one of the largest groups of Ascomycota. This taxonomically highly diverse group plays an important role in nutrient and carbon cycling, biological diversity and ecosystem functioning. The diversity of lignicolous freshwater fungi along a north-south latitudinal gradient is currently being studied in Asia. In this paper, we introduce two novel freshwater taxa viz. *Tingoldiago hydei***sp. nov.** and *T. clavata***sp. nov.** which were collected from freshwater substrates in Eastern Thailand. Morphological comparison based on the size of ascomata, asci and ascospores, as well as multi-gene phylogenetic analyses based on LSU, SSU, ITS and TEF1-α DNA sequences, supports their placement in *Tingoldiago* (Lentitheciaceae). Descriptions and illustrations of these two new species are provided.

## Introduction

Freshwater fungi are those which the whole or part of their life cycle is found in a freshwater habitat (Thomas 1996, Wong et al. 1998) and they are an evolutionary important group (Vijaykrishna et al. 2006). The members of freshwater fungi can be saprobes, parasites, endophytes and mutualistic taxa (Vijaykrishna et al. 2005, Zhang et al. 2008, Swe et al. 2009, Jones et al. 2014, Huang et al. 2018). There is a wide range of organisms that can be freshwater fungi hosts, such as wood, plants, alga, foams, fish etc. (Sparrow 1960, Ellis and Ellis 1985, Jones et al. 2014). However, a lot of studies on freshwater fungi have focused on lignicolous freshwater fungi (Tsui et al. 2000, Cai et al. 2002, Luo et al. 2004, 2018, Jones et al. 2014, Hyde et al. 2016, Yang et al. 2017), which were defined as those fungi that grow on submerged woody debris in freshwater streams, ponds, lakes and tree hollows (Hyde et al. 2016). They also grow on submerged wood in peat swamps and dams (Pinnoi et al. 2006, Pinruan et al. 2007, 2014, Hu et al. 2010). Lignicolous freshwater fungi are a diverse group comprising species from different phyla (Aphelidiomycota, Ascomycota, Basidiomycota, Blastocladiomycota, Chytridiomycota, Monoblepharomycota, Mortierellomycota and Rozellomycota) (Shearer et al. 2007, Kagami et al. 2012, Zhang et al. 2012, Jones et al. 2014, Wijayawardene et al. 2018). The dominant groups of lignicolous freshwater fungi are Dothideomycetes and Sordarialmycets (Jones et al. 2014, Hyde et al. 2016, Wijayawardene et al. 2017, 2018).

We are studying the diversity of lignicolous freshwater fungi in Thailand, in order to establish the phylogenetic relationships of lignicolous freshwater fungi, understanding the natural classification of this group and contributing to the biogeographical diversity of fungi (Hyde et al. 2016). The study on freshwater fungi in Thailand was first investigated by Tubaki et al. (1983) and they reported 40 freshwater fungal species from foam. Subsequently, mycologists started to study lignicolous freshwater fungi in Thailand and several taxa have been reported (Sivichai et al. 1998, 1999, 2000, 2002, 2010, Jones et al. 1999, Marvanová et al. 2000, Hu et al. 2010, Zhang et al. 2013, Luo et al. 2015, 2016, Bao et al. 2018).

Lentitheciaceae was introduced by Zhang et al. (2012) to accommodate *Massarina*-like species in the order Pleosporales. Presently, 13 genera are accepted in this family (Dayarathne et al. 2018, Hyde et al. 2018). Species in this family are widely distributed in the world (China, Egypt, Hungary, Italy, Japan, Russia, Saudi, Thailand, UK, Uzbekistan) and are commonly saprobic on stems and twigs of herbaceous and woody plants in terrestrial or aquatic habitats (Wanasinghe et al. 2014, 2018, Knapp et al. 2015, Wijayawardene et al. 2015, Luo et al. 2016, Tibpromma et al. 2017, Hyde et al. 2018). The genus *Tingoldiago* was established by Hirayama et al. (2010) with a single species *Tingoldiago graminicola* K. Hiray. & Kaz. Tanak, this species being originally treated as *Massarina ingoldiana*. Later, Hirayama et al. (2010) re-assessed the phylogeny of *Massarina ingoldiana* and introduced two new genera *Tingoldiago and Lindgomyces* to accommodate *Massarina ingoldianasensu lato*, based on phylogenetic analyses. Currently, only one species is accepted in this genus.

In this paper, we introduce two new freshwater species of *Tingoldiago* (Lentitheciaceae), based on morpho-molecular studies. Detailed descriptions and illustrations of these two new species are provided.

## Materials and methods

### Collection, Isolation and morphological studies

Submerged decaying wood samples were collected from That Phanom, Nakhon Phanom, Thailand and brought to the laboratory in plastic bags. The samples were incubated in plastic boxes lined with moistened tissue paper at room temperature for one week. Specimen observations and morphological studies were conducted, following the protocols provided by Luo et al. (2018).

Pure cultures were obtained by single spore isolation followed by Chomnunti et al. (2014). Germinating ascospores were transferred aseptically to potato dextrose agar (PDA) plates and grown at 16–25 °C in daylight. Colony colour and other characters were observed and measured after three weeks. The specimens were deposited in the herbarium of Mae Fah Luang University (MFLU), Chiang Rai, Thailand. Living cultures are deposited in the Culture Collection of Mae Fah Luang University (MFLUCC). Facesoffungi numbers and Index Fungorum numbers were obtained, following Jayasiri et al. (2015) and Index Fungorum (2019). New species have been established as recommended by Jeewon and Hyde (2016).

### DNA extraction, PCR amplification and sequencing

Fungal mycelium was scraped from the surface of colonies grown on a PDA plate or MEA plate at 25 °C for 4 weeks, transferred into a 1.5 ml centrifuge tube and ground using liquid nitrogen. The EZ geneTM fungal gDNA kit (GD2416) was used to extract DNA from the ground mycelium according to the manufacturer’s instructions. The gene regions of the large subunit of the nuclear ribosomal DNA (LSU), the internal transcribed spacers (ITS), the small subunit of the nuclear ribosomal DNA (SSU) and the translation elongation factor (TEF1-α) RNA were amplified using the primer pairs LR0R/LR7 (Vilgalys and Hester 1990), ITS5/ITS4, NS1/ NS4 (White et al. 1990) and 983F/2218R (Liu et al. 1999), respectively. The amplification reactions were performed in 25 μl of PCR mixtures containing 9.5 μl ddH_2_O, 12.5 μl 2× PCR MasterMix (Tsingke Co., China), 1 μl DNA sample and 1μl of each primer. The PCR thermal cycle programme for LSU, ITS, SSU and TEF1-α amplification were as follows: 94 °C for 3 minutes, followed by 35 cycles of denaturation at 94 °C for 30 seconds, annealing at 56 °C for 50 seconds, elongation at 72 °C for 1 minute and a final extension at 72 °C for 10 minutes and finally kept at 4 °C. PCR amplification was confirmed on 1% agarose electrophoresis gels stained with ethidium bromide. PCR products were sequenced using the same set of primers used in PCR in Beijing Tsingke Biological Engineering Technology and Services Co. Ltd. (Beijing, P.R. China).

### Sequencing and sequence alignment

The sequence was assembled by using BioEdit and sequences with high similarity indices were determined from a BLAST search to find the closest matches with taxa in Lentitheciaceae and from recently published data (Dayarathne et al. 2018). All consensus sequences and the reference sequences were aligned using MAFFT v. 7 (http://mafft.cbrc.jp/alignment/server/index.html) (Katoh and Standley 2013), then checked visually and manually optimised using BioEdit v.7.0.9 (Hall 1999). Ambiguous regions were excluded from the analyses and gaps were treated as missing data. The phylogeny website tool “ALTER” (Glez-Peña et al. 2010) was used to convert the alignment fasta file to Phylip format for RAxML analysis and Clustalx BETA and PAUP 4.0 were used to convert the alignment fasta file to a Nexus file for Bayesian analysis. Phylogenetic analyses were obtained from Maximum Likelihood (ML), Maximum Parsimony (MP) and Bayesian analysis.

### Phylogenetic analyses

**Figure 1.**
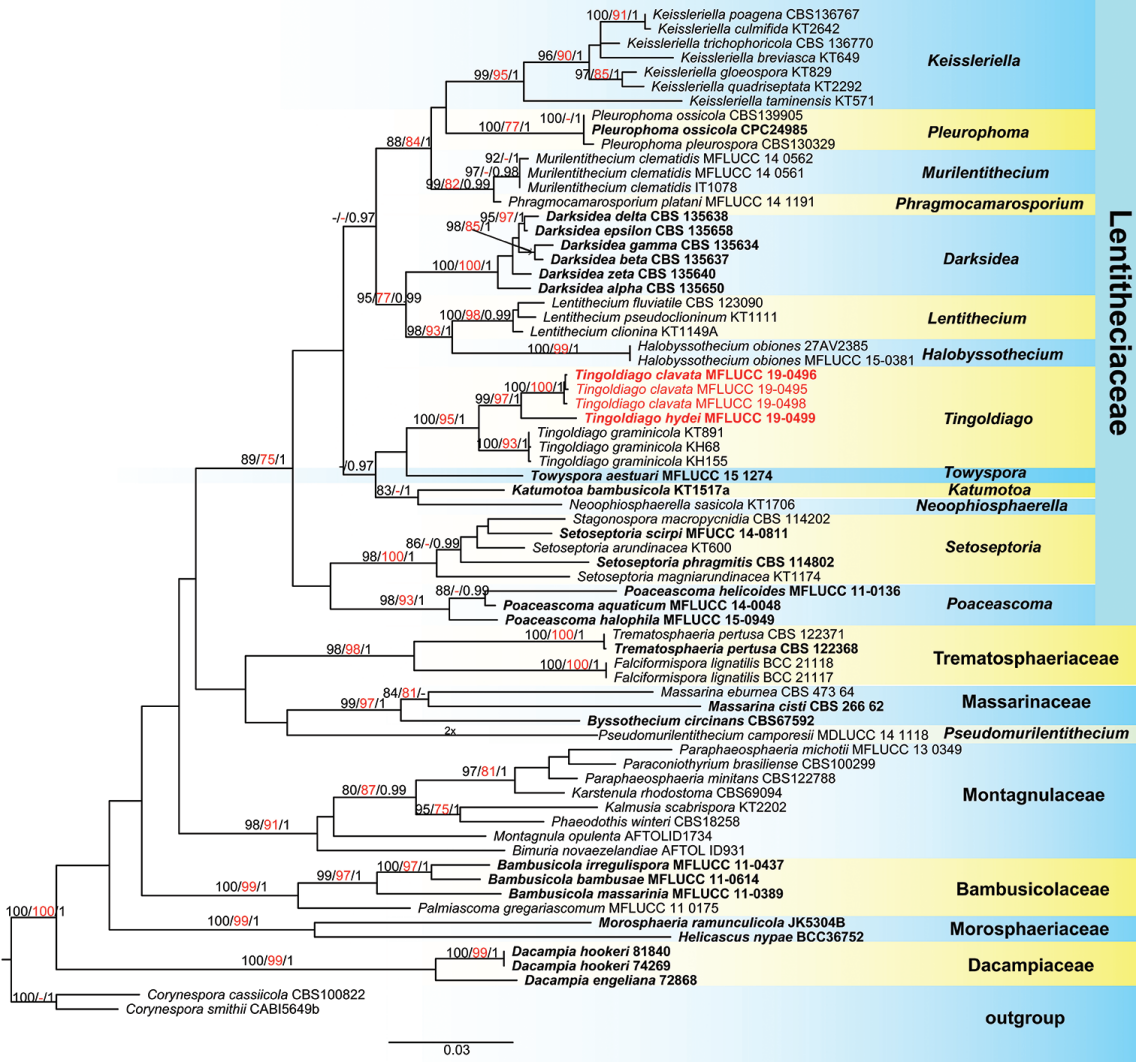
Phylogenetic tree based on RAxML analyses of combined LSU, SSU, ITS and TEF1-α sequence data. Bootstrap support values for maximum likelihood (ML, black) and maximum parsimony (MP, red) higher than 75% and Bayesian posterior probabilities (PP, black) greater than 0.95 are indicated above the nodes as MP / ML /PP. The ex-type strains are in bold and the newly obtained isolates are in red. The tree is rooted at *Corynespora smithii* (CABI5649b) and *Corynespora cassiicola* (CBS100822).

Maximum likelihood trees were generated using the RAxML-HPC2 on XSEDE (8.2.8) (Stamatakis 2006, Stamatakis et al. 2008) in the CIPRES Science Gateway platform (Miller et al. 2010) using GTR+ I + G model of evolution which was estimated by MrModeltest 2.2 (Nylander et al. 2008). Maximum likelihood bootstrap values (ML), equal to or greater than 75%, are given above each node (Figure 1).

MP analyses were performed using the heuristic search option with 1000 random taxa addition and tree bisection and reconnection (TBR) as the branch-swapping algorithm. All characters were unordered and of equal weight and gaps were treated as missing data. Maxtrees were unlimited, branches of zero length were collapsed and all multiple, equally parsimonious trees were saved. Clade stability was assessed using a bootstrap (BS) analysis with 1000 replicates, each with ten replicates of random stepwise addition of taxa (Hillis and Bull 1993).

The Bayesian analysis was performed with MrBayes v3.2 (Ronquist et al. 2012), with the best-fit model of sequence evolution estimated with MrModeltest 2.2 (Nylander et al. 2008) to evaluate posterior probabilities (PP) (Rannala and Yang 1996, Zhaxybayeva and Gogarten 2002) by Markov Chain Monte Carlo (MCMC) sampling. Six simultaneous Markov chains were run for 10,000,000 generations, trees were sampled every 1000^th^ generation and 1,0000 trees were obtained. Based on the tracer analysis, the first 1,000 trees representing 10% were discarded as the burn-in phase in the analysis. The remaining trees were used to calculate posterior probabilities in the majority rule consensus tree (critical value for the topological convergence diagnostic set to 0.01).

The phylograms were visualised in FigTree 1.4.2 (Rambaut 2014) and made in Adobe Illustrator CS5 (Adobe Systems Inc., USA). All newly generated sequences of this study have been submitted in GenBank.

**Table 1.** Taxa used in this study and their GenBank accession numbers, the newly generated sequences are indicated wirh * and the type strains are indicated in bold.

Taxa	strain	GenBank accession number			
		LSU	SSU	ITS	TEF1
***Bambusicola bambusae***	**MFLUCC 11–0614**	**JX442035**	**JX442039**	**NR121546**	**KP761722**
***B. irregulispora***	**MFLUCC 11–0437**	**JX442036**	**JX442040**	**NR121547**	**KP761723**
***B. massarinia***	**MFLUCC 11–0389**	**JX442037**	**JX442041**	**NR121548**	–
*Bimuria novaezelandiae*	AFTOL ID931	–	–	–	DQ471087
***Byssothecium circinans***	**CBS67592**	**GU205217**	**GU205235**	–	**GU349061**
*Corynespora cassiicola*	CBS100822	GU301808	GU296144	–	GU349052
*C. smithii*	CABI5649b	GU323201	–	–	GU349018
***Dacampia engeliana***	**72868**	**KT383791**	–	–	–
***D. hookeri***	**74269**	**KT383793**	–	–	–
***D. hookeri***	**81840**	**KT383795**	–	–	–
***Darksidea alpha***	**CBS 135650**	**KP184019**	**KP184049**	**NR137619**	**KP184166**
***D. beta***	**CBS 135637**	**KP184023**	**KP184049**	**NR137957**	**KP184189**
***D. delta***	**CBS 135638**	–	–	**NR137075**	–
***D. epsilon***	**CBS 135658**	**KP184029**	**KP184070**	**NR137959**	**KP184186**
***D. gamma***	**CBS 135634**	**KP184031**	**KP184073**	**NR137587**	**KP184188**
***D. zeta***	**CBS 135640**	**KP184013**	**KP184071**	**NR137958**	**KP184191**
*Falciformispora lignatilis*	BCC 21117	GU371826	GU371834	KF432942	GU371819
*F. lignatilis*	BCC 21118	GU371827	GU371835	KF432943	GU371820
*Halobyssothecium obiones*	27AV2385	–	–	KX263864	–
*H. obiones*	MFLUCC 15–0381	MH376744	MH376745	MH377060	MH376746
***Helicascus nypae***	**BCC36752**	**GU479789**	**GU479755**	–	**GU479855**
*Kalmusia scabrispora*	KT2202	AB524594	AB524453	–	AB539107
*Karstenula rhodostoma*	CBS69094	GU301821	GU296154	–	GU349067
***Katumotoa bambusicola***	**KT1517a**	**AB524595**	**AB524454**	**LC014560**	**AB539108**
*Keissleriella breviasca*	KT649	AB807588	AB797298	–	AB808567
*K. culmifida*	KT2642	AB807592	AB797302	LC014562	–
*K. gloeospora*	KT829	AB807589	AB797299	LC014563	–
*K. poagena*	CBS136767	KJ869170	–	KJ869112	–
*K. quadriseptata*	KT2292	AB807593	AB797303	AB811456	AB808572
*K. taminensis*	KT571	AB807595	AB797305	LC014564	AB808574
*K. trichophoricola*	CBS 136770	KJ869171	–	KJ869113	–
*Lentithecium clionina*	KT1149A	AB807540	AB797250	LC014566	AB808515
***L. fluviatile***	**CBS 123090**	**FJ795450**	**FJ795492**	–	–
*L. pseudoclioninum*	KT1111	AB807544	AB797254	AB809632	AB808520
***Massarina cisti***	**CBS 266 62**	**FJ795447**	**FJ795490**	**LC014568**	**AB808514**
*M. eburnea*	CBS 473 64	GU301840	GU296170	–	GU349040
*Montagnula opulenta*	AFTOLID1734	DQ678086	AF164370	–	–
***Morosphaeria ramunculicola***	**JK5304B**	**GU479794**	**GU479760**	–	–
*Murilentithecium clematidis*	IT1078	KM408758	KM408760	KM408756	–
*M. clematidis*	MFLUCC 14–0562	KM408759	KM408761	KM408757	KM454445
*Neoophiosphaerella sasicola*	KT1706	AB524599	AB524458	LC014577	AB539111
*Palmiascoma gregariascomum*	MFLUCC 11–0175	KP744495	KP753958	KP744452	–
*Paraconiothyrium brasiliense*	CBS100299	JX496124	AY642523	JX496011	–
*Paraphaeosphaeria michotii*	MFLUCC 13–0349	KJ939282	KJ939285	KJ939279	–
*P. minitans*	CBS122788	EU754173	EU754074	–	GU349083
*Phaeodothis winteri*	CBS18258	–	GU296183	–	–
*Phragmocamarosporium platani*	MFLUCC 14–1191	KP842915	KP842918	–	–
*Pleurophoma ossicola*	CBS139905	KR476769	–	KR476736	–
***P. ossicola***	**CPC24985**	**KR476770**	–	**NR137992**	–
*Pleurophoma pleurospora*	CBS130329	JF740327	–	–	–
***Poaceascoma aquaticum***	**MFLUCC 14–0048**	**KT324690**	**KT324691**	–	–
***P. halophila***	**MFLUCC 15–0949**	**MF615399**	**MF615400**	–	–
***P. helicoides***	**MFLUCC 11–0136**	**KP998462**	**KP998463**	**KP998459**	**KP998461**
***Pseudomurilentithecium camporesii***	**MDLUCC 14-1118**	**MN638846**	**MN638850**	**MN638861**	–
*Setoseptoria arundinacea*	KT600	AB807575	AB797285	LC014595	AB808551
*S. magniarundinacea*	KT1174	AB807576	AB797286	LC014596	AB808552
***S. phragmitis***	**CBS 114802**	**KF251752**	–	**KF251249**	–
***S. scirpi***	**MFUCC 14–0811**	**KY770982**	**KY770980**	**MF939637**	**KY770981**
*Stagonospora macropycnidia*	CBS 114202	GU301873	GU296198	–	GU349026
*Tingoldiago graminicola*	KH155	AB521745	AB521728	LC014599	AB808562
*T. graminicola*	KH68	AB521743	AB521726	LC014598	AB808561
*T. graminicola*	KT891	AB521744	AB521727	–	AB808563
*T. hydei	**MFLUCC 19-0499**	**MN857177**	–	**MN857181**	–
*T. clavata	**MFLUCC 19-0496**	**MN857178**	**MN857186**	**MN857182**	–
*T. clavata	MFLUCC 19-0498	MN857179	MN857187	MN857183	–
*T. clavata	MFLUCC 19-0495	MN857180	MN857188	MN857184	–
***Towyspora aestuari***	**MFLUCC 15–1274**	**KU248852**	**KU248853**	**NR148095**	–
***Trematosphaeria pertusa***	**CBS 122368**	**FJ201990**	**FJ201991**	**NR132040**	**KF015701**
*Trematosphaeria pertusa*	CBS 122371	GU301876	GU348999	KF015669	KF015702

## Results

### Phylogenetic analyses

The aligned sequence matrix comprises LSU, SSU, ITS and TEF1-α sequence data for 69 taxa, with *Corynespora smithii* and *Corynespora cassiicola* as out-group taxa. The dataset comprises 3334 characters after alignment including gaps (LSU: 1–897; SSU: 898–1920; ITS: 1921–2522; TEF1-α: 2523–3479). The topologies of RAxML, MP and Bayesian are similar and the bootstrap support values for Maximum Likelihood (ML), Maximum Parsimony (MP) higher than 75% and Bayesian posterior probabilities (PP) greater than 0.95 are given above the nodes. Maximum parsimony analyses indicated that 2,442 characters were constant, 232 variable characters parsimony uninformative and 805 characters are parsimony-informative. The RAxML analysis of the combined dataset yielded the best scoring tree (Figure 1) with a final ML optimisation likelihood value of -21568.713178. The matrix had 1322 distinct alignment patterns, with 30.89% undetermined characters or gaps. Estimated base frequencies were as follows: A = 0.238228, C = 0.248262, G = 0.272670, T = 0.240839; substitution rates AC = 1.161111, AG = 2.490274, AT = 1.596115, CG = 1.194931, CT = 7.261814, GT = 1.000000; gamma distribution shape parameter α = 0.183824.

The novel species *Tingoldiago hydei* and *T. clavata*, introduced in this paper, are supported by multi-phylogenetic analyses. Four newly generated strains clustered together within *Tingoldiago* with strong statistical support (100 ML/95 MP/1.00 PP, Figure. 1). Three strains of *T. clavata* clustered together and sister to *T. hydei* with strong bootstrap support (99 ML/97 MP/1 PP, Figure 1).

## Taxonomy

### Tingoldiago hydei

Taxon classificationFungiPleosporalesLentitheciaceae

D.F. Bao, Z.L. Luo & H.Y. Su sp. nov.

71CF27F5-B5F2-5627-8728-FAA5BBB81D1B

Index Fungorum No: IF557047

Facesoffungi No: FoF07082

Figure 2

#### Etymology.

Referring to Kevin D. Hyde for his contributions in fungal taxonomy.

#### Holotype.

Thailand, That Phanom, Nakhon Phanom, on submerged decaying wood, 13 November 2018, D.F. Bao, B-126 (MFLU 19–2842, holotype), ex-type living culture, MFLUCC 19–0499.

#### Description.

*Saprobic* on submerged decaying wood. ***Sexual morph***: *Ascomata* 180–280 × 330–470 μm (*x̄* = 400 × 420 μm, n = 10), immersed to semi-immersed, erumpentia, gregarious, scattered, depressed globose to conical with a flattened base, dark brown to black, as dark spots on host surface. *Ostioles* central, papillate, short, crest-like, dark brown. *Peridium* 33.5–50 μm wide, comprising 4–6 layers, brown to dark brown cells of *textura anngularis*. *Hamathecium* comprising 2–2.5 μm (n = 30) wide, numerous, branched, septate, hyaline, cellular pseudoparaphyses. *Asci* 95–164 × 18–22 μm (*x̄* = 129 × 20 μm, n = 20), 8-spored, bitunicate, fissitunicate, cylindrical-clavate, rounded at apex, with a short pedicellate. *Ascospores* 37.5–42 × 7.5–9 μm (*x̄* = 40 × 8 μm, n = 30), overlapping, 2–3-seriate, clavate with round ends, straight, uniseptate, deeply constricted at septum, with broad and short upper cells 17.5–20 × 7–8.7 μm (*x̄* = 18.7 × 7.9 μm, n = 30), narrow and long lower cells 20.6–23.3 × 5.9–7.4 μm (*x̄* = 21.9 × 6.7 μm, n = 30), tapering towards the end, with short appendages at the septum, hyaline, guttulate, smooth, surrounded by a fusiform gelatinous sheath. ***Asexual morph***: Undetermined.

**Figure 2.**
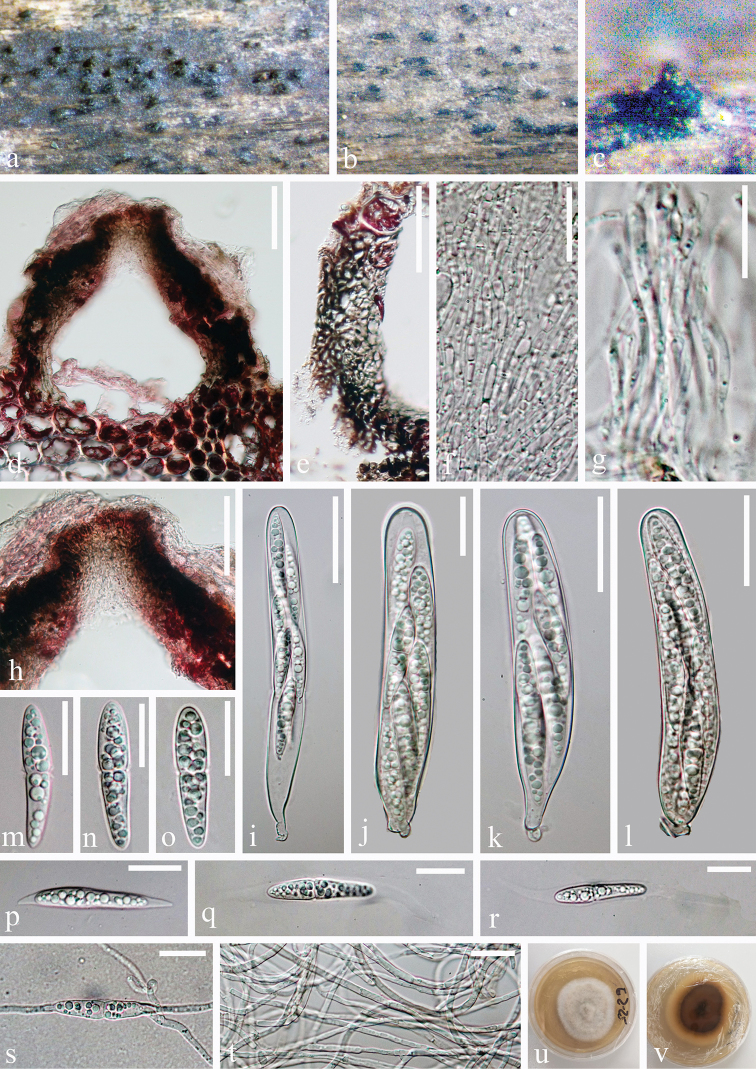
*Tingoldiago hydei* (MFLU 19–2842, holotype). **a–c** Ascomata on wood **d** section of ascoma **e** peridium **f, g** pseudoparaphyses **h** ostiole **i–l** asci **m–r** ascospores **s** germinating ascospore **t** vegetative hyphae in culture **u, v** culture on PDA from surface and reverse. Scale bars: 50 μm (**d, e, h**), 20 μm (**f–g, m–t**), 30 μm (**i–l**).

#### Culture characteristics.

Ascospores germinating on PDA within 24 hours. Colonies on MEA effuse, greyish-white to dark brown from above and below, reaching 3–4 cm diameter within 30 days at room temperature under natural light, composed of subhyaline to pale brown, septate, smooth hyphae.

#### Notes.

Phylogenetic analysis showed that *Tingoldiago hydei* is related to *T. clavata*; however, they are in different lineages with significant support (99 ML/97 MP/1.00 PP, Figure 1). *Tingoldiago hydei* resembles *T. clavata* in having bitunicate, cylindrical-clavate asci and clavate, hyaline, uniseptate, ascospores with broad and short upper cells, narrow and long lower cells, tapering towards the end, surrounded by a gelatinous sheath. However, *Tingoldiago hydei* can be distinguished from *T. clavata* in having longer and narrower asci (95–164 × 18–22 vs. 110–148 × 20–27 μm) and smaller ascospores (37.5–42 × 7.5–9 vs. 48–51 × 7.5–8.5 μm). Moreover, ascospores of *T. clavata* have longer appendages at the septum, while the appendages of *T. hydei* are much shorter than *T. hydei*.

*Tingoldiago clavata* is similar to the type species, *T. raminicola* in having immersed to semi-immersed, depressed globose to conical ascomata with flattened base, bitunicate, fissitunicate, cylindrical-clavate asci and clavate, straight, uniseptate ascospores. However, *T. clavata* differs from *T. raminicola* in having longer asci (95–164 × 18–22 vs. 87.5–122 × 18.25–25 μm) and smaller ascospores (37.5–42 × 7.5–9 vs. 43.5–53 × 7.5–11 μm). Moreover, ascopores of *T. clavata* have short appendages at the septum while ascospores of *T. raminicola* lack appendages. In addition, we compared the base pairs of ITS regions between these two species and there were 25 base pairs without gaps (5.1%) differences. Therefore, we introduce our isolate as a new species based on both phylogeny and morphological characters.

### Tingoldiago clavata

Taxon classificationFungiPleosporalesLentitheciaceae

D.F. Bao, L. Xu & H.Y. Su sp. nov.

2905DCA7-92E5-5C62-A22E-AE1058358E0E

Index Fungorum No: IF557048

Facesoffungi No: FoF07083

Figure 3

#### Etymology.

Referring to the clavate ascospores of this fungus.

#### Holotype.

Thailand, That Phanom, Nakhon Phanom, on submerged decaying wood, 13 November 2018, D.F. Bao, B-161 (MFLU 19–2843, holotype), ex-type culture, MFLUCC 19–0496.

#### Description.

*Saprobic* on submerged decaying wood. ***Sexual morph***: *Ascomata* 145–210 × 145–195 μm (*x̄* = 175 × 169 μm, n = 10), immersed to semi-immersed, gregarious, scattered, erumpentia, depressed globose to conical with a flattened base, dark brown to black, as dark spots on host surface. *Ostiole* central, round to papillate, short, crest-like, dark brown. *Peridium* 28–47 μm wide, comprising several layers, pale brown to brown cells of *textura anngularis*. *Hamathecium* comprising 1.5–2.0 μm (n = 30) wide, numerous, branched, septate, hyaline, cellular pseudoparaphyses. *Asci* 110–148 × 20–27 μm (*x̄* = 129 × 23 μm, n = 20), 8-spored, bitunicate, fissitunicate, cylindrical-clavate, rounded at apex, with a short pedicellate. *Ascospores* 48–51 × 7.5–9 μm (*x̄* = 50.5 × 8.5 μm, n = 30), overlapping, 2–3-seriate, clavate, with round ends, straight, uniseptate, deeply constricted at septum, hyaline, with broad and short upper cells 16.6–18.9 × 7.8–9.0 μm (*x̄* = 17.7 × 8.4 μm, n = 30), narrow and long lower cells 30–32.9 × 6.5–8.0 μm (*x̄* = 31.5 × 7.3 μm, n = 30), tapering towards the end, guttulate, smooth, 2–4 equatorial appendages at the septum and surrounded by a fusiform gelatinous, sheath. ***Asexual morph***：Undetermined.

**Figure 3.**
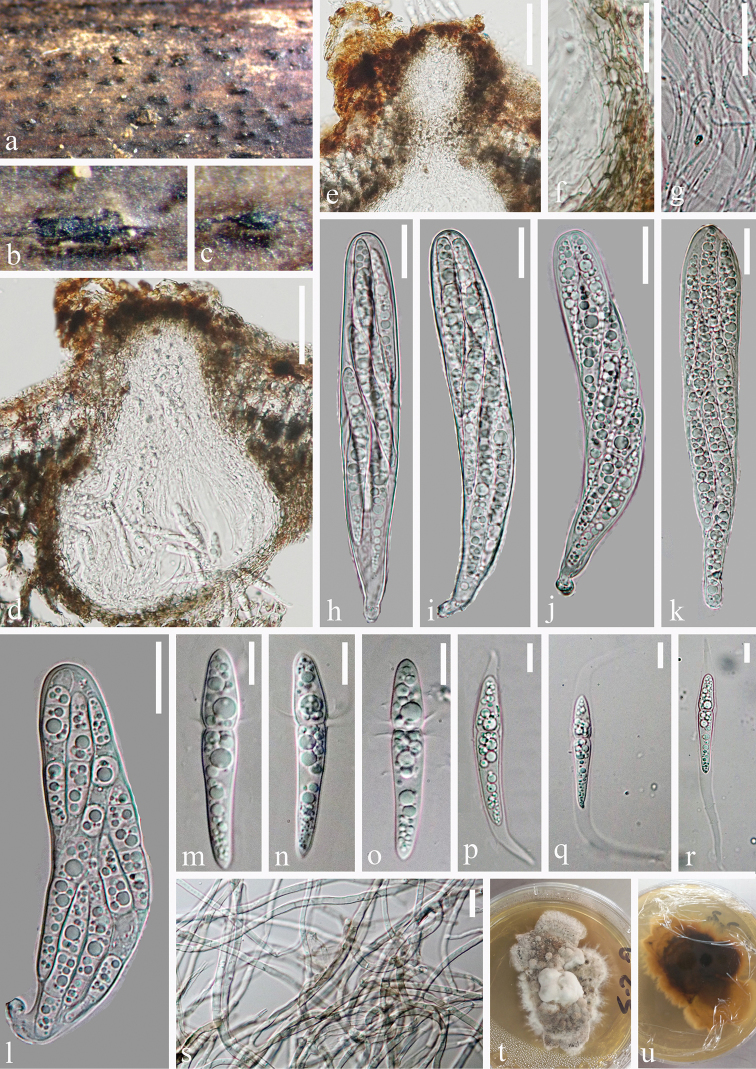
*Tingoldiago clavata* (MFLU 19–2843, holotype). **a–c** ascomata on wood **d** section of ascoma **e** ostiole **f** peridium **g** pseudoparaphyses **h–l** asci **m–r** ascospores **s** vegetative hyphae in culture **t, u** culture on PDA from surface and reverse. Scale bars: 50 μm (**d, e**), 20 μm (**f–l**), 10 μm (**m–s**).

#### Culture characteristics.

Ascospores germinating on PDA within 24 hours. Colonies on MEA effuse, velvety, greyish-white to dark brown from above and below, reaching 2.5–3 cm diameter within 30 days at room temperature under natural light, composed of subhyaline to brown, septate, smooth hyphae.

#### Additional specimens examined.

Thailand, That Phanom, Nakhon Phanom, on submerged decaying wood, 13 November 2018, D.F. Bao, B160 (paratype: MFLU 19–2844; living culture, MFLUCC 19–0498); Thailand, That Phanom, Nakhon Phanom, on submerged decaying wood, 13 November 2018, D.F. Bao, B136 (paratype: MFLU 19–2845; living culture, MFLUCC 19–0495)

#### Notes.

*Tingoldiago clavata* resembles the type species, *T. graminicola* in having bitunicate, cylindrical-clavate asci with a short pedicellate and clavate, hyaline, 1-septate, ascospores with broad upper cells, narrow lower cells. However, we can distinguish them by the size of ascomata and asci and the colour, septate and appendages of ascospores. *Tingoldiago clavata* has smaller ascomata (110–148 ×145–195 vs. 150–250 × 250–450 μm) and larger asci (110–148 × 20–27 vs. 87.5–122 × 18.25–25 μm). Moreover, ascopsores of *T. clavata* are hyaline, uniseptate, with 2–4 equatorial appendages at the septum, while ascopspores of *T. graminicola* are brown and 3-septate at maturity and lacking appendages at the septum. In addition, a comparison of the 491 nucleotides across the ITS gene region of *T. clavata* and *T. graminicola* reveals 25 base-pair differences and therefore provides further evidence to introduce *T. clavata* as a new species as recommended by Jeewon and Hyde (2016).

## Discussion

During the last decade, freshwater fungi in Thailand have been mainly reported from north, south and northeast of Thailand (Jones et al. 1999, Marvanová and Hywel-Jones 2000, Sivichai and Boonyuen 2010, Sivichai and Hywel-Jones 1999, Sivichai et al. 1998, 2000, Sri-indrasutdhi et al. 2010). No freshwater fungi from Eastern Thailand have been reported so far. In this study, two new freshwater species, viz. *Tingoldiago hydei* and *T. clavata* from Eastern Thailand, are introduced, based on morphology and phylogeny. *Tingoldiago hydei* and *T. clavata* satisfied the generic concept of the genus *Tingoldiago* (Hirayama et al. 2010). They comprise globose to conical, immersed to erumpent ascomata, cellular pseudoparaphyses, bitunicate, fissitunicate asci and clavate ascospores with a median primary septum and a large fusiform gelatinous sheath around the ascospore (Hirayama et al. 2010). Morphologically, *T. hydei* and *T. clavata* are quite similar as they have similar shape of asci and ascospores; however, we can distinguish them by the size of ascomata, asci and ascospores (Table 2). In addition, we also compared the morphological differences of these two species with the type species, *T. graminicola*. Ascopores of *T. hydei* and *T. clavata* are hyaline, uniseptate, with appendages at the septum and the upper cells are broader and shorter than the lower cells, while the ascopsores of *T. graminicola* are hyaline, uniseptate, but becoming brown and 3-septate with age, lacking appendages at the septum, upper cells and lower cells are similar lengths. Phylogenetic analyses showed that our two new isolates clustered together and are sister to the type species, *Tingoldiago graminicola* with strong bootstrap support (100 ML/92 MP/1.00 PP). This evidence strongly supports our two isolates to be the new species.

**Table 2.** The morphological comparisons of *Tingoldiago* species discussed in this study.

Taxa	Distribution	Ascomata (μm)	Pseudoparaphyses (μm)	Asci (μm)	Ascospores (μm)	References
*Tingoldiago graminicola*	Japan, UK	150–250 × 250–450	1.5–4	87.5–122 × 18.25–25	43.5–53 × 7.5–11	Hirayama et al. 2010
*T. hydei*	Thailand	180–280 × 330–470	1.8–2.5	95–164 × 18–22	37.5–42 × 7.5–9	This study
*T. clavata*	Thailand	145–210 × 145–195	1.4–2.0	110–148 × 20–27	48–51 × 7.5–8.5	This study

Hyde et al. (2020) introduced a new genus, *Pseudomurilentithecium* in Lentitheciaceae. In their phylogenetic analysis, *Pseudomurilentithecium* clustered with *Poaceascoma* and was basal to Lentitheciaceae. However, in our phylogenetic analysis, *Pseudomurilentithecium* grouped with the members of Massarinaceae, rather than Lentitheciaceae. Therefore, further investigation is required to confirm the placement of the genus.

*Tingoldiago* is a well-resolved genus in this family with a stable clade within Lentitheciaceae. The genus can be distinguished from other genera in this family by having hyaline, uniseptate, upper cells are broad and basal cells are narrow ascospores with a large fusiform gelatinous sheath. The sheath is considered to be an adaptation by the genus that enables ascospores to attach to the substrates in moving water (Shearer 1993, Hyde and Goh 2003, Jones 2006, Devadatha et al. 2019). It is reported that the genus *Tingoldiago* is exclusively found in freshwater habitats (Hirayama et al. 2010) and our two new species were collected from lotic habitats of Mekong River.

## Supplementary Material

XML Treatment for Tingoldiago hydei

XML Treatment for Tingoldiago clavata

## References

[B1] Bao DF Luo ZL Jeewon R Nalumpang S Su HY Hyde KD (2018) *Neoastrosphaeriella aquatica* sp. nov. (Aigialaceae), a new species from freshwater habitat in southern Thailand. Phytotaxa 391: 197–206. 10.11646/phytotaxa.391.3.3

[B2] Cai L Tsui CKM Zhang KQ Hyde KD (2002) Aquatic fungi from Lake Fuxian, Yunnan, China. Fungal Diversity 9: 57–70.

[B3] Dayarathne MC Hyde KD Wanasinghe DN Jones EBG Chomnunti P (2018) A novel marine genus, *Halobyssothecium* (Lentitheciaceae) and epitypification of *Halobyssothecium obiones* comb. nov. Mycological Progress 17: 1161–1171. 10.1007/s11557-018-1432-3

[B4] Devadatha B Sarma VV Jeewon R Hyde KD Jones EBG (2019) *Morosphaeria muthupetensis* sp.nov. (Morosphaeriaceae) from India: Morphological characterisation and multigene phylogenetic inference. Botanica Marina 61: 395–405. 10.1515/bot-2017-0124

[B5] Ellis MB Ellis JP (1985) Microfungi on Land Plants: An Identification Handbook (1^st^ ed.). Macmillan Pub Co.

[B6] Glez-Peña D Gómez-Blanco D Reboiro-Jato M Fdez-Riverola F Posada D (2010) ALTER: program-oriented conversion of DNA and protein alignments. Nucleic Acids Research 38: 14–18. 10.1093/nar/gkq321 PMC2896128 20439312

[B7] Hall TA (1999) BioEdit: a user-friendly biological sequence alignment editor and analysis program for Windows 95/98/NT. Nucleic Acids Symposium Series 41: 95–98. 10.1093/nar/gkq321

[B8] Hillis DM Bull JJ (1993) An empirical test of bootstrapping as a method for assessing confidence in phylogenetic analysis. Systematic Biology 42: 182–192. 10.1093/sysbio/42.2.182

[B9] Hirayama K Tanaka K Raja HA Miller AN Shearer CA (2010) A molecular phylogenetic assessment of *Massarina ingoldiana**sensu lato.* Mycologia 102: 729–746. 10.3852/09-230 20524604

[B10] Hu DM Cai L Chen H Bahkali AH Hyde KD (2010) Fungal diversity on submerged wood in a tropical stream and an artificial lake. Biodiversity and Conservation 19: 3799–3808. 10.1007/s10531-010-9927-5

[B11] Hyde KD (1995) Tropical Australia freshwater fungi VII. New genera and species of ascomycetes. Nova Hedwigia 61: 119–140.

[B12] Hyde KD Chaiwan N Norphanphoun C Boonmee S Camporesi E Chethana KWT Dayarathne MC de Silva NI Dissanayake AJ Ekanayaka AH Hongsanan S Huang SK Jayasiri SC Jayawardena RS Jiang HB Karunarathna A Lin CG Liu JK Liu NG Lu YZ Luo ZL Maharachchikumbura SSN Manawasinghe IS Pem D Perera RH Phukhamsakda C Samarakoon MC Senwanna C Shang QJ Tennakoon DS Thambugala KM Tibpromma S Wanasinghe DN Xiao YP Yang J Zeng XY Zhang JF Zhang SN Bulgakov TS Bhat DJ Cheewangkoon R Goh TK Jones EBG Kang JC Jeewon R Liu ZY Lumyong S Kuo CH Mckenzie EHC Wen TC Yan JY Zhao Q (2018) Mycosphere notes 169–224. Mycosphere 9(2): 271–430. 10.5943/mycosphere/9/2/8

[B13] Hyde KD Fryar S Tian Q Bahkali AH Xu JC (2016) Lignicolous freshwater fungi along a north-south latitudinal gradient in the Asian/Australian region; can we predict the impact of global warming on biodiversity and function? Fungal Ecology 19: 190–200. 10.1016/j.funeco.2015.07.002

[B14] Hyde KD Goh TK (2003) Adaptations for dispersal in filamentous freshwater fungi. Fungal Diversity 10: 231–258.

[B15] Hyde KD Jones EBG Liu JK Ariyawansa H Boehm E Boonmee S Braun U Chomnunti P Crous PW Dai DQ Diederich P Dissanayake A Doilom M Doveri F Hongsanan S Jayawardena R Lawrey JD Li YM Liu YX Lücking R Monkai J Muggia L Nelsen MP Pang KL Phookamsak R Senanayake IC Shearer CA Suetrong S Tanaka K Thambugala KM Wijayawardene NN Wikee S Wu HX Zhang Y Aguirre-Hudson B Alias SA Aptroot A Bahkali AH Bezerra JL Bhat DJ Camporesi E Chukeatirote E Gueidan C Hawksworth DL Hirayama K Hoog SD Kang JC Knudsen K Li WJ Li XH Liu ZY Mapook A McKenzie EHC Miller AN Mortimer PE Phillips AJL Raja HA Scheuer C Schumm F Taylor JE Tian Q Tibpromma S Wanasinghe DN Wang Y Xu JC Yacharoen S Yan JY Zhang M (2013) Families of Dothideomycetes. Fungal Diversity 63: 1–313. 10.1007/s13225-013-0263-4

[B16] Hyde KD Dong Y Phookamsak R Jeewon R Bhat DJ Jones EBG Liu NG Abeywickrama PD Mapook A Wei DP Perera RH Manawasinghe IS Pem D Bundhun D Karunarathna A Ekanayaka AH Bao DF Li JF Samarakoon MC Chaiwan N Lin CG Phutthacharoen K Zhang SN Senanayake IC Goonasekara ID Thambugala KM Phukhamsakda C Tennakoon DS Jiang HB Yang J Zeng M Huanraluek N Liu JK Wijesinghe SN Tian Q Tibpromma S Brahmanage RS Boonmee S Huang SK Thiyagaraja V Lu YZ Jayawardena LS Dong W Yang EF Singh SK Singh SM Rana S Lad SS Anand G Devadatha B Niranjan M Sarma VV Liimatainen K Aguirre-Hudson B Niskanen T Overall A Alvarenga RLM Gibertoni TB Pliegler WP Horváth E Imre A Alves AL Santos ACDS Tiago RV Bulgakov TS Wanasinghe DN Bahkali AH Doilom M Elgorban AM Maharachchikumbura SSN Rajeshkumar KC Haelewaters D Mortimer PE Zhao Q Lumyong S Xu JC Sheng J (2020) Fungal diversity notes 1151–1276: taxonomic and phylogenetic contributions on genera and species of fungal taxa. Fungal Diversity In press.

[B17] Huang SK Jeewon R Hyde KD Bhat JD Wen TC (2018) Novel taxa within Nectriaceae: *Cosmosporella* gen. nov. and *Aquanectria* sp. nov. from freshwater habitats in China. Crytogamie Mycologie 39: 169–192. 10.7872/crym/v39.iss2.2018.169

[B18] Jayasiri SC Hyde KD Ariyawansa HA Bhat DJ Buyck B Cai L Dai YC Abd-Elsalam KA Ertz D Hidayat I Jeewon R Jones EBG Bahkali AH Karunarathna SC Liu JK Luangsa-ard JJ Lumbsch HT Maharachchikumbura SSN McKenzie EHC Moncalvo JM Ghobad-Nejhad M Nilsson H Pang KA Pereira OL Phillips AJL Raspé O Rollins AW Romero AI Etayo J Selçuk F Stephenson SL Suetrong S Taylor JE Tsui CKM Vizzini A Abdel-Wahab MA Wen TC Boonmee S Dai DQ Daranagama DA Dissanayake AJ Ekanayaka AH Fryar SC Hongsanan S Jayawardena RS Li WJ Perera RH Phookamsak R de Silva NI Thambugala KM Tian Q Wijayawardene NN Zhao RL Zhao Q. Kang JC Promputtha I (2015) The faces of fungi database: fungal names linked with morphology, phylogeny and human impacts. Fungal Diversity 74: 3–18. 10.1007/s13225-015-0351-8

[B19] Jeewon R Hyde KD (2016) Establishing species boundaries and new taxa among fungi: recommendations to resolve taxonomic ambiguities. Mycosphere 7: 1669–1677. 10.5943/mycosphere/7/11/4

[B20] Jones EBG (2006) Form and function of fungal spore appendages. Mycoscience 47: 167–183. 10.1007/S10267-006-0295-7

[B21] Jones EBG Hyde KD Pang KL (2014) Freshwater Fungi and Fungal-like Organisms. De Gruyter, Germany. 10.1515/9783110333480

[B22] Jones EBG Wong SW Sivichai S Au DWT Hywel-Jones NL (1999) Lignicolous freshwater ascomycota from Thailand: *Micropeltopsis quinquecladiopsis* sp. nov. Mycological Research 103: 729–735. 10.1017/S0953756298007618

[B23] Kagami M Amano Y Ishii N (2012) Community structure of planktonic fungi and the impact of parasitic chytrids on phytoplankton in Lake Inba, Japan. Microbial Ecology 63: 358–368. 10.1007/s00248-011-9913-9 21805083

[B24] Katoh K Standley DM (2013) MAFFT multiple sequence alignment software version 7: improvements in performance and usability. Molecular Biology and Evolution 30: 772–780. 10.1093/molbev/mst010 23329690 PMC3603318

[B25] Knapp DG Kovács GM Zajta E Groenewald JZ Crous PW (2015) Dark septate endophytic pleosporalean genera from semiarid areas. Persoonia 35: 87–100. 10.3767/003158515X687669 26823630 PMC4713113

[B26] Liu YJ Whelen S Hall BD (1999) Phylogenetic relationships among ascomycetes: evidence from an RNA polymerase II subunit. Molecular Biology and Evolution 16: 1799–1808. 10.1093/oxfordjournals.molbev.a026092 10605121

[B27] Luo J Yin JF Cai L Zhang KQ Hyde K D (2004) Freshwater fungi in Lake Dianchi, a heavily polluted lake in Yunnan, China. Fungal Diversity 16: 93–112.

[B28] Luo ZL Maharachchikumnura SSN Liu XY Li SH Chen LJ Su HY Zhou DQ Hyde KD (2015) *Annulatascus saprophyticus* sp. nov. and *Pseudoannulatascus* gen. nov. to accommodate *Annulatascus biatriisporus* (AnnulatascalesSordariomycetes) from Thailand. Phytotaxa 239(2): 174–182. 10.11646/phytotaxa.239.2.6

[B29] Luo ZL Bahkali AH Liu XY Phookamsak R Zhao YC Zhou DQ Su HY Hyde KD (2016) *Poaceascoma aquaticum* sp. nov. (Lentitheciaceae), a new species from submerged bamboo in freshwater. Phytotaxa 253(1): 71–80. 10.11646/phytotaxa.253.1.5

[B30] Luo ZL Hyde KD Liu JK Bhat DJ Bao DF Li WL Su HY (2018) Lignicolous freshwater fungi from China II: Novel *Distoseptispora* (Distoseptisporaceae) species from northwestern Yunnan Province and a suggested unified method for studying lignicolous freshwater fungi. Mycosphere 9: 444–461. 10.5943/mycosphere/9/3/2

[B31] Marvanová L Hywel-Jones NL (2000) *Sigmoidea conforta* sp. nov. and two rare hyphomycete species from streams in Thailand. Cryptogamie Mycologie 21: 13–26. 10.1016/S0181-1584(00)00101-9

[B32] Miller MA Pfeiffer W Schwartz T (2010) Creating the CIPRES Science Gateway for inference of large phylogenetic trees. Proceedings of the 2010 Gateway Computing Environments Workshop (GCE): 1–8. 10.1109/GCE.2010.5676129

[B33] Nylander JAA Wilgenbusch JC Warren DL Swofford DL (2008) AWTY (are we there yet?): a system for graphical exploration of MCMC convergence in Bayesian phylogenetics. Bioinformatics 24: 581–583. 10.1093/bioinformatics/btm388 17766271

[B34] Pinnoi A Lumyong S Hyde KD Jones EBG (2006) Biodiversity of fungi on the palm *Eleiodoxa conferta* in Sirindhorn peat swamp forest, Narathiwat, Thailand. Fungal Diversity 22: 205–218.

[B35] Pinruan U Lumyong S Hyde KD Jones EBG (2007) Occurrence of fungi on tissues of the peat swamp palm *Licuala longecalycata*. Fungal Diversity 25: 157–173.

[B36] Pinruan U Lumyong S Hyde KD Jones EBG (2014) Tropical peat swamp fungi with special reference to palms. In: Jones EBG Hyde KD Pang KL (Eds) Freshwater Fungi.De Gruyter, Germany, 371–388. 10.1515/9783110333480.371

[B37] Rambaut A (2014) FigTree v1.4: tree figure drawing tool. http://tree.bio.ed.ac.uk/software/figtree/

[B38] Rannala B Yang Z (1996) Probability distribution of molecular evolutionary trees: a new method of phylogenetic inference. Journal of Molecular Evolution 43(3): 304–311. 10.1007/BF02338839 8703097

[B39] Ronquist F Teslenko M van der Mark P Ayres DL Darling A Höhna S Larget B Liu L Suchard MA Huelsenbeck JP (2012) MrBayes 3.2: efficient Bayesian phylogenetic inference andmodel choice across a large model space. Systematic Biology 61(3): 539–542. 10.1093/sysbio/sys029 22357727 PMC3329765

[B40] Sheaer CA (1993) The freshwater ascomycetes. Nova Hedwigia 56: 1–33.

[B41] Shearer CA Descals E Kohlmeyer B Kohlmeyer J Marvanová L Padgett D Porter D Raja HA Schmit JP Thorton HA Voglmayr H (2007) Fungi biodiversity in aquatic habitats. Biodiversity and Conservation 16: 49–67. 10.1007/s10531-006-9120-z

[B42] Sivichai S Boonyuen N (2010) *Jahnula morakotii* sp. nov. and *J. appendiculata* from a peat swamp in Thailand. Mycotaxon 112: 475–481. 10.5248/112.475

[B43] Sivichai S Hywel-Jones N (1999) *Biflagellospora* (aero-aquatic hyphomycetes) from submerged wood in Thailand. Mycological Research 103: 908–914. 10.1017/S0953756298007928

[B44] Sivichai S Hywel-jones NL Jones EBG (1998) Lignicolous freshwater Ascomycota from Thailand: 1. *Ascotaiwania sawada* and its anamorph state Monotosporella. Mycoscience 39: 307–311. 10.1007/BF02464013

[B45] Sivichai S Hywel-jones NL Somrithipol S (2000) Lignicolous freshwater Ascomycota from Thailand: *Melanochaeta* and *Sporoschisma anamorph*s. Mycological Research 104: 478–485. 10.1017/S0953756299001604

[B46] Sivichai S Jones EBG Hywel-Jones NL (2000) Fungal colonisation of wood in a freshwater stream at Khao Yai National Park, Thailand. Fungal Diversity 5: 71–88.

[B47] Sivichai S Jones EBG Hywel-Jones NL (2002) Fungal colonisation of wood in a freshwater stream at Tad Ta Phu, Khao Yai National Park, Thailand. Fungal Diversity 10: 113–129.

[B48] Sparrow FK (1960) Aquatic Phycomycetes (Second Rev). The University of Michigan Press, Ann Arbor. 10.5962/bhl.title.5685

[B49] Sri-indrasutdhi V Boonyuen N Suetrong S Chuaseeharonnachai C Sivichai S Jones EBG (2010) Wood-inhabiting freshwater fungi from Thailand: *Ascothailandia grenadoidia* gen. et sp. nov., *Canalisporium grenadoidia* sp. nov. with a key to *Canalisporium* species (Sordariomycetes, Ascomycota). Mycoscience 51: 411–420. 10.1007/S10267-010-0055-6

[B50] Stamatakis A (2006) RAxML-VI-HPC: maximum likelihood-based phylogenetic analyses with thousands of taxa and mixed models. Bioinformatics 22(21): 2688–2690. 10.1093/bioinformatics/btl446 16928733

[B51] Stamatakis A Hoover P Rougemont J (2008) A rapid bootstrap algorithm for the RAxML web servers. Systematic Biology 57: 758–771. 10.1080/10635150802429642 18853362

[B52] Swe A Jeewon R Pointing SB Hyde KD (2009) Diversity and abundance of nematode-trapping fungi from decaying litter in terrestrial, freshwater and mangrove habitats. Biodiversity and Conservation 18: 1695–1714. 10.1007/s10531-008-9553-7

[B53] Thomas K (1996) Australian freshwater fungi. In: Grgurinovic CA (Ed.) Introductory Volume to the Fungi (Part2). Fungi of Australian. Canberra, Australia: Australian Biological Resources Study 1–37.

[B54] Tibpromma S Hyde KD Jeewon R Maharachchikumbura SSN Liu JK Bhat DJ Jones EBG McKenzie EHC Camporesi E Bulgakov TS Doilom M de Azevedo Santiago ALCM Das K Manimohan P Gibertoni TB Lim YW Ekanayaka AH Thongbai B Lee HB Yang JB Kirk PM Sysouphanthong P Singh SK Boonmee S Dong W Raj KNA Latha KPD Phookamsak R Phukhamsakda C Konta S Jayasiri SC Norphanphoun C Tennakoon DS Li JF Dayarathne MC Perera RH Xiao Y Wanasinghe DN Senanayake IC Goonasekara ID de Silva NI Mapook A Jayawardena RS Dissanayake AJ Manawasinghe IS Chethana KWT Luo ZL Hapuarachchi KK Baghela A Soares AM Vizzini A Meiras-Ottoni A Mešić A Dutta AK de Souza CAF Richter C Lin CG Chakrabarty D Daranagama DA Lima DX Chakraborty D Ercole E Wu F Simonini G Vasquez G da Silva GA Plautz HL Ariyawansa HA Lee H Kušan I Song J Sun JZ Karmakar J Hu K Semwal KC Thambugala KM Voigt K Acharya K Rajeshkumar KC Ryvarden L Jadan M Hosen MI Mikšík M Samarakoon MC Wijayawardene NN Kim NK Matočec N Singh PN Tian Q Bhatt RP de Oliveira RJV Tulloss RE Aamir S Kaewchai S Marathe SD Khan S Hongsanan S Adhikari S Mehmood T Bandyopadhyay TK Svetasheva TY Nguyen TTT Antonín V Li WJ Wang Y Indoliya Y Tkalčec Z Elgorban AM Bahkali AH Tang AMC Su HY Zhang H Promputtha I Luangsaard J Xu JC Yan JY Kang JC Stadler M Mortimer PE Chomnunti P Zhao Q Phillips AJL Nontachaiyapoom S Wen T-C Karunarathna SC (2017) Fungal diversity notes 491–602: taxonomic and phylogenetic contributions to fungal taxa. Fungal Diversity 83(1): 1–261. 10.1007/s13225-017-0378-0

[B55] Tsui CKM Hyde KD Hodgkiss IJ (2000) Biodiversity of fungi on submerged wood in Hong Kong streams. Aquatic Microbial Ecology 21: 289–298. 10.3354/ame021289

[B56] Tubaki K Watanabe K Manoch L (1983) Aquatic hyphomycetes from Thailand. Transactions of the Mycological Society of Japan 24: 451–457. 10.3354/ame021289

[B57] Vilgalys R Hester M (1990) Rapid genetic identification and mapping of enzymatically amplified ribosomal DNA from several Cryptococcus species. Journal of Bacteriology 172: 4238–4246. 10.1128/JB.172.8.4238-4246.1990 2376561 PMC213247

[B58] Vijaykrishna D Jeewon R Hyde KD (2005) *Fusoidispora aquatica*: New freshwater ascomycetes from Hong Kong based on morphology and molecules. Sydowia 57: 267–280.

[B59] Vijaykrishna D Jeewon R Hyde KD (2006) Molecular taxonomy, origins and evolution of freshwater ascomycetes. Fungal Diversity 23: 367–406.

[B60] Wanasinghe DN Jones EBG Camporesi E Boonmee S Ariyawansa HA Wijayawardene NN Hyde KD (2014) An Exciting Novel Member of Lentitheciaceae in Italy from Clematis Vitalba. Cryptogamie Mycologie 35(4): 323–337. 10.7872/crym.v35.iss4.2014.323

[B61] Wanasinghe DN Phukhamsakda C Hyde KD Jeewon R Lee HB Jones EG Tibpromma S Tennakoon DS Dissanayake AJ Jayasiri SC GafforovErio Y Camporesi E Bulgakov TS Ekanayake AH Perera RH Samarakoon SC Goonasekara ID Mapook A Li WJ Senanayake IC Li JF Norphanphoun C Doilom M Bahkali AH Xu JC Mortimer PE Tibell L Tibell S Karunarathna SC (2018) Fungal diversity notes 709–839: taxonomic and phylogenetic contributions to fungal taxa with an emphasis on fungi on Rosaceae. Fungal Diversity 89(1): 1–236. 10.1007/s13225-018-0395-7

[B62] White TJ Bruns T Lee S Taylor J (1990) Amplification and direct sequencing of fungal ribosomal RNA genes for phylogenetics. In: Innis GM Shinsky D White T (Eds) PCR protocols: a guide to methods and applications.Academic, New York, 315–322. 10.1016/B978-0-12-372180-8.50042-1

[B63] Wijayawardene NN Hyde KD Bhat DJ Goonasekara ID Nadeeshan D Camporesi E Schumacher RK Yong W (2015) Additions to Brown Spored Coelomycetous Taxa in Massarinae, Pleosporales: Introducing *Phragmocamarosporium* gen. nov. and *Suttonomyces* gen. nov. Cryptogamie Mycologie 36: 213–224. 10.7872/crym/v36.iss2.2015.213

[B64] Wijayawardene NN Hyde KD Rajeshkumar KC Hawksworth DL Madrid H Kirk PM Braun U Singh RV Crous PW Kukwa M Lűcking R Kurtzman CP Yurkov A Haelewaters D Aptroot A Lumbsch HT Timdal E Ertz D Etayo J Phillips AJL Groenewald JZ Papizadeh M Selbmann L Dayarathne MC Weerakoon G Jones EBG Suetrong S Tian Q Castanéda-Ruiz RF Bahkali AH Pang KL Tanaka K Dai DQ Sakayaroj J Hujslová M Lombard L Shenoy BD Suija A Maharachchikumbura SSN Thambugala KM Wanasinghe DN Sharma BO Gaikwad S Pandit G Zucconi L Onofri S Egidi E Raja HA Kodsueb R Cáceres MES Pérez-Ortega S Fiuza PO Monteiro JS Vasilyeva LN Shivas RG Prieto M Wedin M Olariaga I Lateef AA Agrawal Y Fazeli SAS Amoozegar MA Zhao GZ Pﬂiegler WP Sharma G Oset M Abdel MA Takamatsu S Bensch K Silva NI De Kesel A Karunarathna A Boonmee S Pﬁster DH Lu YZ Luo ZL Boonyuen N Daranagama DA Senanayake IC Jayasiri SC Samarakoon MC Zeng XY Doilom M Quijada L Rampadarath S Heredia G Dissanayake AJ Jayawardana RS Perera PH Tang LZ Phukhamsakda C Hernández-Restrepo M Ma XY Tibpromma S Gusmao LFP Weerahewa D Karunarathna SC (2017) Notes for genera: Ascomycota. Fungal Diversity 86: 1–594. 10.1007/s13225-017-0386-0

[B65] Wijayawardene NN Pawłowska J Letcher PM Kirk PM Humber RA Schüßler A Wrzosek M Muszewska A Okrasińska A Istel Ł Gęsiorska A Mungai P Lateef AA Rajeshkumar KC Singh RV Radek R Walther G Wagner L Walker C Wijesundara DSA Papizadeh M Dolatabadi S Shenoy BD Tokarev YS Lumyong S Hyde KD (2018) Notes for genera: basal clades of Fungi (including Aphelidiomycota, Basidiobolomycota, Blastocladiomycota, Calcarisporiellomycota, Caulochytriomycota, Chytridiomycota, Entomophthoromycota, Glomeromycota, Kickxellomycota, Monoblepharomycota, Mortierellomycota, Mucoromycota, Neocallimastigomycota, Olpidiomycota, Rozellomycota and Zoopagomycota). Fungal Diversity 92: 43–129. 10.1007/s13225-018-0409-5

[B66] Wong MKM Goh TK Hodgkiss IJ Hyde KD Ranghoo VM Tsui CKM Ho WH Wong WSW Yuen TK (1998) Role of fungi in freshwater ecosystems. Biodiversity and Conservation 7: 1187–1206. 10.1023/A:1008883716975

[B67] Yang J Liu JK Hyde KD Jones EBG Liu Z (2017) Two new species in Fuscosporellaceae from freshwater habitat in Thailand. Mycosphere 8: 1893–1903. 10.5943/mycosphere/8/10/12

[B68] Zhang H Jones EBG Zhou DQ Bahkali AH Hyde KD (2011) Checklist of freshwater fungi in Thailand. Cryptogamie, Mycologie 32: 199–217. 10.7872/crym.v32.iss2.2011.199

[B69] Zhang H Hyde KD Abdel-Wahab MA Abdel-Aziz F A Ariyawansa HA KoKo TW Zhao RL Alias SA Bahkali AH Zhou DQ (2013) A modern concept for *Helicascus* with a *Pleurophomopsis*-like asexual state. Sydowia 65: 147–166.

[B70] Zhang Y Jeewon R Fournier J Hyde KD (2008) Multi-gene phylogeny and morphotaxonomy of *Amniculicola lignicola*: novel freshwater fungus from France and its relationships to the Pleosporales. Fungal Biology 112: 1186–1194. 10.1016/j.mycres.2008.04.004 18783929

[B71] Zhang Y Crous PW Schoch CL Hyde KD (2012) Pleosporales. Fungal Diversity 53: 1–221. 10.1007/s13225-011-0117-x 23097638 PMC3477819

[B72] Zhaxybayeva O Gogarten JP (2002) Bootstrap, Bayesian probability and maximum likelihood mapping: exploring new tools for comparative genome analyses. BMC Genomics 3: 4. 10.1186/1471-2164-3-4 PMC100357 11918828

